# Investigation of Laser-Induced Periodic Surface Structures Using Synthetic Optical Holography

**DOI:** 10.3390/nano12030505

**Published:** 2022-02-01

**Authors:** Krisztian Neutsch, Evgeny L. Gurevich, Martin R. Hofmann, Nils C. Gerhardt

**Affiliations:** 1 Photonics and Terahertz Technology, Ruhr-Universität Bochum, Universitätsstraße 150, 44801 Bochum, Germany; martin.hofmann@rub.de (M.R.H.); nils.gerhardt@rub.de (N.C.G.); 2 Applied Laser Technology, Ruhr-Universität Bochum, Universitätsstraße 150, 44801 Bochum, Germany; gurevich@lat.rub.de; 3 Laser Center (LFM), University of Applied Sciences Münster, Stegerwaldstraße 39, 48565 Steinfurt, Germany

**Keywords:** synthetic optical holography, LIPSS

## Abstract

In this paper, the investigation of laser-induced periodic surface structures (LIPSSs) on a polycrystalline diamond substrate using synthetic optical holography (SOH) is demonstrated. While many techniques for LIPSS detection operate with sample contact and/or require preparation or processing of the sample, this novel technique operates entirely non-invasively without any processing of or contact with the LIPSS sample at all. The setup provides holographic amplitude and phase images of the investigated sample with confocally enhanced and diffraction-limited lateral resolution, as well as three-dimensional surface topography images of the periodic structures via phase reconstruction with one single-layer scan only.

## 1. Introduction

Laser-induced periodic surface structures (LIPSSs) are periodic patterns formed on surfaces of different materials (metals [1,2,3], semiconductors [4,5], and dielectrics [6]) exposed to laser radiation. They were discovered by Birnbaum in 1965 [4], who observed the periodic damage pattern induced by a ruby laser on a semiconductor with a conventional optical microscope. These three-dimensional surface patterns are used to functionalize surfaces for a number of applications. While some typical examples are the structural colorization [7,8] and texturing [9,10] of surfaces, LIPSSs can also be used to modify surface wettability [11,12], for biological applications such as anti-bacterial surfaces [13,14], and surface-enhanced Raman scattering (SERS-EFs) [15,16].

While LIPSS-related research topics have concentrated mainly on the generation and application of these structures, the tools for their study are very limited and remain mostly outside the main research focus. Usually, standard visualization techniques available in the laboratory have been applied to characterize LIPSSs. For CO${}_{2}$-laser-induced periodic patterns with spacing in the range 10–20 μm on highly polished metallic surfaces, dark-field microscopy was used to increase the pattern contrast [1]. Now, high-power, short- or ultrashort-pulse lasers in near-IR or visible range of wavelengths are usually used for LIPSS generation. Since the period of the emerging patterns is usually less than the wavelength of the laser light, the application of either high-resolution optical microscopes or other surface visualization techniques with sub-micrometer resolution is mandatory.

Scanning electron microscopy (SEM) [2,3,17] and atomic-force microscopy (AFM) [18] have become more and more common in LIPSS studies due to their higher lateral resolution. On the one hand, electron microscopy can be combined with other methods for chemical analysis, such as energy-dispersive X-ray (EDX) [19]; it also provides some information about the chemical composition (e.g., oxidation) of the pattern. On the other hand, direct access to reliable 3D patterns is not possible. Moreover, SEM requires conductive surfaces for the electron beam interaction. In cases of non-conductive samples, e.g., bioorganic specimens or dielectric materials, coating of the surface with a metallic layer is usually required, which irreversibly modifies the surface chemistry of the sample [17]. Furthermore, the chemical composition can influence the local electric conductivity, which leads to local charge accumulation and additional scattering of the incident electron beam. In this way, the surface conductivity pattern can interfere with the topographic information and distort the SEM image. However, the three-dimensional structure of the LIPSS can be acquired with electron microscopy only if surface imaging is combined with sample cutting or slicing. The cross section of the LIPSS visualized with transmission electron microscopy (TEM) can extend the SEM image to give an impression of the three-dimensional pattern structure, especially if multilayered samples are used in the experiments [20]. A three-dimensional structure can be also reconstructed in several cycles; in each of them, the sample is first polished and etched down to a certain depth, then imaged with an electron microscope [21]. This technique is highly invasive and time consuming, and requires the destruction of the sample. On the other hand, three-dimensional imaging of LIPSSs using AFM operates by interacting with the sample’s surface; therefore, it requires direct mechanical surface access, with its tip extremely close or even in contact with the sample.

In order to enable a non-destructive investigation, all-optical methods are used to visualize LIPSS patterns, such as white-light interferometry (WLI) [22] or confocal laser scanning microscopy [23]. However, these methods are either limited to two-dimensional intensity images only or require axial translation of the sample in order to fully capture the three-dimensional pattern on the surface. None of them is capable of three-dimensional imaging with only one two-dimensional measurement.

In this paper, we present a new all-optical, non-invasive method for three-dimensional visualization of LIPSS patterns exploiting the benefits of digital holographic microscopy (DHM). Our approach to investigate LIPSSs is based on the principle of synthetic optical holography (SOH) [24] and combines synthetic holographic imaging with a confocal laser-scanning microscope. While this new technique has been shown to provide high-quality amplitude and phase images [25,26,27,28], its capabilities for the investigation of LIPSS have not yet been explored. Although the setup is based on laser-scanning confocal interferometry, which is inherently in-line, this approach provides all the advantages of off-axis holography. The setup performs a two-dimensional line-by-line scan of the sample and, by adding a synthetic reference wave, holographic image acquisition is enabled. The reconstruction of the scanned hologram provides high-resolution amplitude and phase images, as well as three-dimensional surface plots of the sample.

This technique operates without any mechanical contact with the sample; does not require any preparation, handling or treatment; and entirely preserves the sample in its original condition. It provides amplitude images based on the distribution of back-reflected intensities with a diffraction-limited lateral resolution of about 600 nm for an illumination wavelength of 1064 nm. In contrast to other techniques typically used for imaging LIPSSs, this technique additionally provides refractive index- and height-sensitive phase images of the sample, from which 3D topographic surface images can be extracted. Last but not least, the technical and financial effort is lower than that of the other mentioned techniques typically used for imaging LIPSSs. This makes this technique capable of cost-effective investigation of laser-induced periodic surface structures, while fully preserving the sample in its original condition.

## 2. Materials and Methods

### 2.1. Investigated Laser-Induced Periodic Surface Structures

In our experiments, LIPSSs were generated on a polycrystalline diamond plate. The laser pulses (Tangerine fs laser, produced by Amplitude Systems), with λ=1030 nm and pulse duration of 270 fs, were focused on the diamond plate surface with an F-theta lens (focal length of 63 mm and numerical aperture 0.06). The repetition rate of the laser was $f_{L}=200\;kHz$, while the spot size, defined as 1/e intensity width, was $w_{0}\approx 7.5\;\mu$m. The Rayleigh length was $z_{R}\approx 149\;\mu$m. A galvo-scanner (SCAN-cube 7 by SCANLAB) was used to scan the beam over the surface with the scanning velocity v=0.1 m/s. These parameters correspond to a pulse overlap along one line of approximately $N_{eff}=15$ pulses per site.

The laser processing parameters were chosen as follows: the pulse fluence was $F=11\;J/cm^{2}$ and the pulse energy $E_{p}\approx 5\;\mu$J, corresponding to a peak power of $P_{p}\approx 20$ MW. The nonlinear refractive index in CVD polycrystalline diamond was $n_{2}\approx 1.5\times 10^{-15}\;$cm${}^{2}/$W [29], while the linear refractive index was n=2.4, which corresponds to the critical power for self-focusing of $P_{c}=\frac{0.15\lambda^{2}}{nn_{2}}\approx 0.45\;$MW [30], so that the self-focusing in the diamond can assist laser processing.

**Figure 1:**
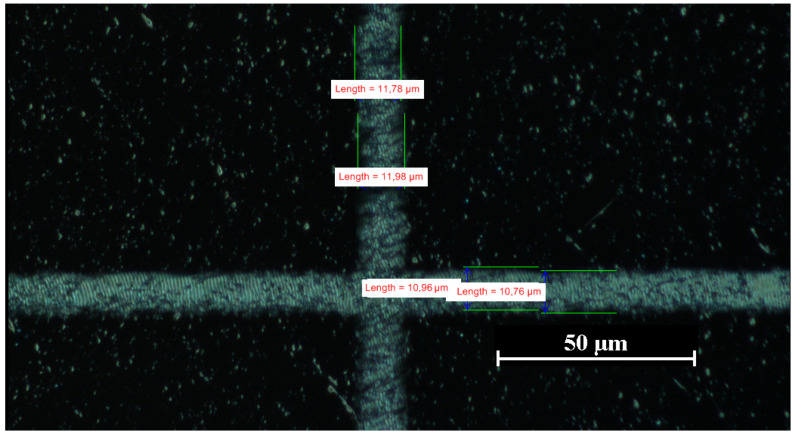
Laser-induced periodic surface structures on the surface of a polycrystalline diamond plate, imaged with a commercial optical microscope (Nikon, ×100) in dark-field illumination mode.

**Figure 2:**
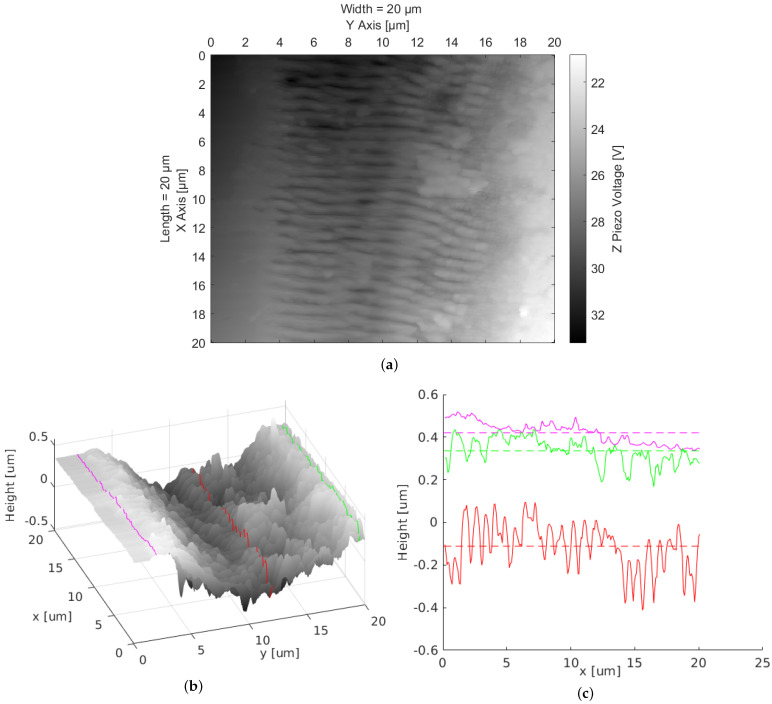
Atomic-force microscope measurement of polycrystalline diamond sample containing laser-induced periodic surface structures: (**a**) 2D image (Z piezo voltage is proportional to surface height); (**b**) 3D image of the same area; (**c**) investigated depth of line profiles along the sample as marked in (**b**) and their mean values (dashed lines).

After laser processing, the LIPSSs were studied with commercially available techniques, as well as with our confocal laser-scanning holographic microscope. For standard techniques, we used a *Nikon Eclipse LV100 microscope* (see Figure 1) and a contact-mode atomic-force microscope *Thorlabs EDU-AFM1/M* (see Figure 2).

The orientation of the LIPSSs is related to the polarization direction in the beam focus. The periodic structures appear almost vertically all over the sample, no matter whether the overall valley structure containing the periodic pattern is oriented horizontally or vertically (see Figure 1). We attribute the tilt of the LIPSSs with respect to the scanning direction in Figure 1 to a galvanometer scanner induced transformation of the originally horizontal polarization of the laser beam to an elliptical one with a position-dependent ratio between the main axes [31]. The surface scan of the LIPSS sample using the AFM (Figure 2) shows an orthogonal pattern of periodic surface structures. By investigating the period length of the structures, accounting for the distances of structure peaks along the red line in the x-direction, an average period length of 0.83μm with a standard deviation of 0.12μm was derived.

### 2.2. Synthetic Optical Holographic Microscope

The holographic imaging of the samples was performed using a confocal laser-scanning microscope setup adapted to apply the principle of synthetic optical holography (SOH) [25,26,27,28]. This method is based on a Michelson interferometer and combines the well-established technique of confocal laser-scanning microscopy [32,33] with the extension of a moving reference mirror in order to enable holographic image acquisition with a synthetic reference wave, which behaves analogously to a plane reference wave in an off-axis holographic setup [25].

**Figure 3:**
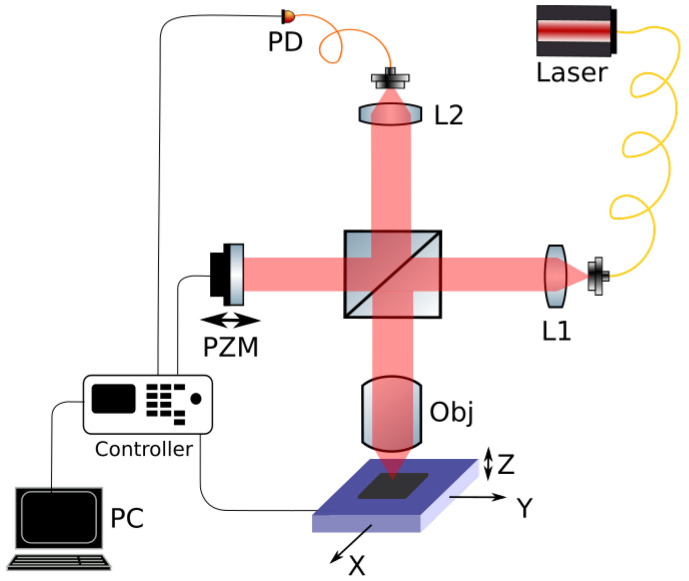
Synthetic optical holographic microscope for imaging of the LIPSS sample: L1, L2—lenses; Obj—microscope objective; PZM—piezo-mounted reference mirror; X, Y, and Z—stage axes; PD—photodiode.

Figure 3 depicts the setup. The beam of a temperature-controlled pigtail diode laser (Lumics SN0687946) with λ=1064 nm was collimated using Lens L1 and directed towards a fifty-fifty beamsplitter, where it was divided into two parts. One part, the object beam, was focused by a long-working-distance high-resolution microscope objective Obj (Leica HC PL FLUOTAR L 100×; NA = 0.75). In the focal plane of the objective, the sample was located and moved by two linear translation stages (Newport M-UMR8.25 + LTA-HL) in order to scan the surface point-by-point. Before scanning, a third stage Z (Newport M-MVN80 + LTA-HL) was used to position the sample into the focal plane of objective Obj.

The other part of the beam, used as reference beam, was directed onto a silver mirror that was placed on a piezo translation stage PZM (Newport NPXYZ100SG). While scanning the sample, the reference mirror was shifted step-wise along the optical axis. The reflections of both beams from the reference and sample arms were coupled into a 62.5 μm graded index multimode fiber via Lens L2 and interfered at the photodiode PD. Due to its diameter, the fiber acted as a pinhole, enabling optical sectioning and causing the confocal behavior of the setup.

At the photodiode, the interference signal was detected and mapped with the sample position known from the scanning stages by the controller (Newport XPS Q8). Although the reference wave has a constant phase for each detected point (pixel), its phase shifting during the scan creates a virtual tilt of the reference plane in the overall image. This virtual tilt between reference and object creates the off-axis behavior [25], although there is no physical off-axis here, and this is a pure in-line configuration. Still, the strong advantages of off-axis holography can be exploited, such as angular frequency filtering due to separation of image orders, thus avoiding the twin-image problem during reconstruction [34]. Moreover, the step size of the moving reference mirror PZM can be used to directly determine the off-axis angle and, by adjusting this value, the reference plane can be “synthesized” as needed to sufficiently separate the image orders in the spectrum.

After the interference of the virtually tilted reference beam and the object beam from the sample was captured by photodetector PD, it was amplified with a transimpedance amplifier and converted by the analog-digital converter of the controller. Due to the confocal imaging approach, the measured intensities mapped with the stage positions resulted in an off-axis holographic image containing the scanned sample image superimposed with a holographic interference pattern, from which the three-dimensional wavefront of the sample’s reflection could be numerically reconstructed. Consequently, numerical propagation was not mandatory for the image reconstruction.

This numerical reconstruction was performed using the angular spectrum method [34], spectral filtering of the first order image, and Zernike polynomials for aberration and background tilt correction [34]. The result of the reconstruction is given in amplitude and phase images of the scanned sample. While the amplitude image is formed by the reflected intensity at each point of the sample, the phase image represents phase changes between the light reflected at the surface (object) and the plane reference wavefront. The measured overall phase changes Δφ are caused by height variations $\Delta \varphi_{h}$ as well as reflection-induced phase shifts due to the complex refractive indices $\Delta \varphi_{n}$ of the sample [35,36].

(1) $$\Delta \varphi =\Delta \varphi_{h}+\Delta \varphi_{n}$$

While $\Delta \varphi_{n}$ can be derived from Fresnel’s equations for orthogonal surface reflections [37], the remaining phase difference $\Delta \varphi_{h}$ can be used to identify the structure’s height variation [34]. In cases of phase differences larger than the wavelength (in reflection measurement, half of the wavelength to account for the return path), the 2π-ambiguity causes phase discontinuities. This issue is a well-known phenomenon and commonly addressed using phase-unwrapping algorithms [34], which recognize these discontinuities and compensate for them by adding 2π at the corresponding areas. A tilt of the sample plane versus the image plane also contributes to the measured height difference. However, since the investigated structures are below this height limit, the 2π-ambiguity and the necessity for compensation was prevented in the first place by numerically correcting only the sample tilt. This was achieved by shifting the selected spectral window for reconstruction, which virtually tilts the image plane by such an amount that the physical sample tilt was compensated for and phase discontinuities did not occur.

### 2.3. Setup Characterization

**Figure 4:**
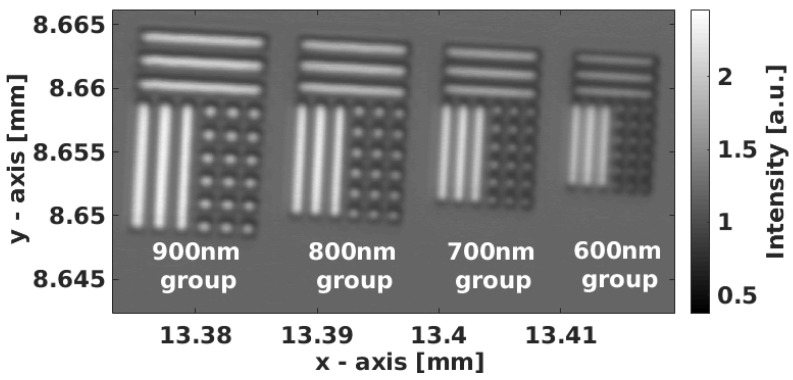
Overview of custom test target. The widths of dots and lines and the distances between are 900 nm, 800 nm, 700 nm, and 600 nm, respective to their group. The image was recorded with our SOH microscope in (non-holographic) confocal mode.

**Figure 5:**
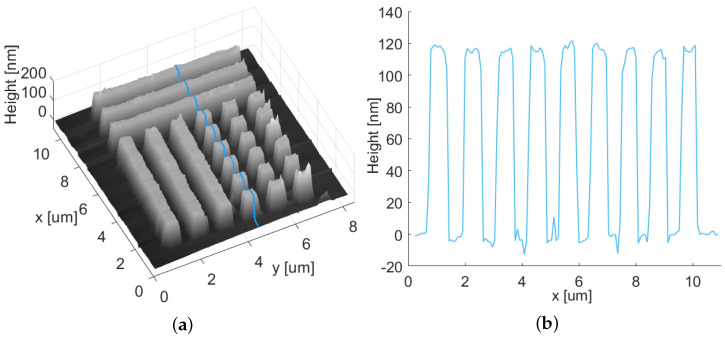
3D reference image (**a**) and height profile (**b**) of the 600 nm group of the test target, showing lateral and height dimensions. This reference image was recorded with an AFM.

The characterization of the setup was performed with a custom test target containing an approximately 120 nm-thick layer of titanium structures on a silicon surface fabricated by electron-beam lithography and evaporation. The Ti structures were grouped in different sizes in sub-micrometer range. The groups contained dots and bars, with the group name indicating the dot width, line width, and distance between the structures. Figure 4 gives an overview of the test target, showing the 900 nm, 800 nm, 700 nm, and 600 nm structure groups. For a detailed overview, Figure 5 shows a reference measurement (a) of the test target’s 600 nm group and the height profile (b) of the indicated line, performed with an AFM.

**Figure 6:**
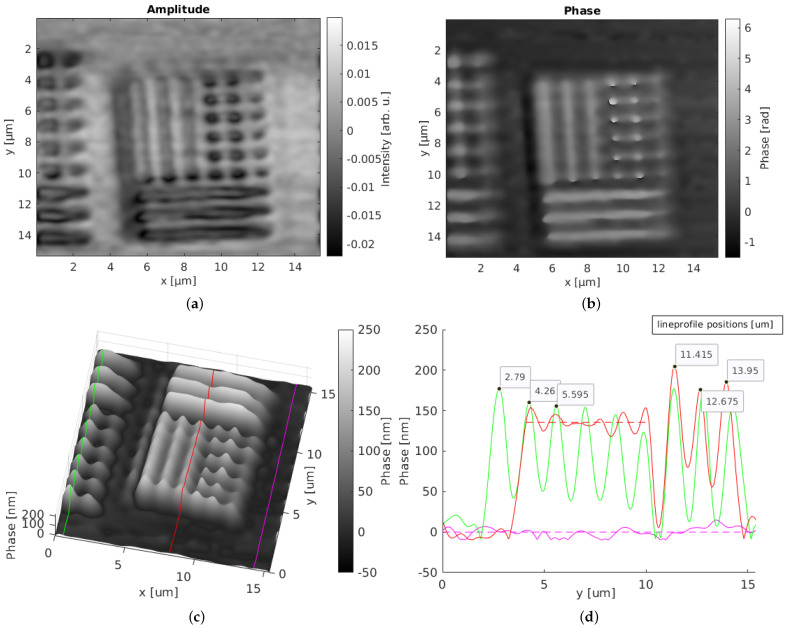
Holographic images of the resolution test target containing structures with line widths and dot sizes of 700 nm (left, partly shown in subfigures (**a**–**c**)) and 600 nm (in the center of the subfigures (**a**–**c**)), recorded with the synthetic optical holographic microscope: (**a**) amplitude image, (**b**) phase image, (**c**) 3D surface plot, and (**d**) phase values of line profiles extracted from (**c**).

The holographic investigation of the test target with our synthetic optical holographic microscope is shown in Figure 6. Figure 6a,b show the reconstructed amplitude and phase images of the 600 nm group (in the center of the images) and parts of the 700 nm group (left, partly shown in the images). Figure 6c depicts the three-dimensional phase map of the same field of view and Figure 6d shows the three extracted line profiles—one taken from the 700 nm group (green line), another one from the 600 nm group (red line), and one from the silicon surface (purple line).

The calculated diffraction limit of the system, according to the Rayleigh criterion together with the enhancement factor $\frac{1}{\sqrt{2}}$ due to confocality of the system, results in a theoretically expected resolution limit of 611 nm [38]. The practically estimated resolution was obtained by imaging structures of size 600 nm on the test target with our confocal laser-scanning holographic microscope, which confirms the diffraction-limited resolution of our system. Furthermore, the separation of adjacent points with group-sized distance between them was evaluated using three structure maxima from the extracted line profile in Figure 6a. The theoretically expected distance between the maxima is double that of the group size. The practically measured distances had an average value of 1.40 μm for the 700 nm structure group and an average distance of 1.27 μm for the 600 nm group, proving the high lateral accuracy of the system for the investigation of laser-induced periodic surface structures and demonstrating the diffraction-limited resolution of our system.

The phase map of the investigated test target provides a topographic surface plot with the three-dimensional shape of the test target structures. The lateral dimensions agree well with the known sample information. In order to derive the height $\Delta \varphi_{h}$, the phase change caused by the complex refractive index difference $\Delta \varphi_{n}$ needed to be subtracted. Based on Fresnel’s equations [37], this phase difference was $\Delta \varphi_{n}=0.288\;rad$, which corresponds to an effective height difference of $\Delta \varphi_{n}=24.7\;nm$. With a closer look at the bar structure of the 600 nm group, the measured phase had an average value of $\bar{\Delta \varphi }=135.7\;nm$ (red dashed line in Figure 6d). Subtracting the refractive index-related phase difference $\Delta \varphi_{n}$, we derived a measured height of $\Delta \varphi_{h}=110.9\;nm$ for the expected ∼120 nm structure height. Considering the background fluctuations in the Si surface (purple line) with a variance of 32 nm, the derived structure height is within the measurement tolerance and proves the system’s capability for quantitative phase measurements. Further measurement inaccuracies may be caused by native oxide layers on top of the Ti and Si structures. They may influence $\Delta \varphi_{h}$ by causing an additional layer to transmit through, which increases the optical path length. Although their influence cannot be quantified here, it can be assumed that they contribute to the overall measurement error.

Additional spikes and exaggerations in the phase values occurred during numerical reconstruction at sharp edges, as only limited spatial frequencies were taken into account during reconstruction. Their influence on the resulting image is the higher the smaller the imaged structures are. This is particularly notable in the 600 nm structures (red line), which are slightly below the diffraction limit where the modulation depth is also limited, which causes uncertainties when imaging strong edges over small areas. In contrast, the adjacent 700 nm structures appear smoother and more accurate. Other factors influencing the deviation of the measured phase height versus the physical structure height could be related to phase noise from the environment and the measurement system itself. Nevertheless, the lateral and axial accuracies were adequate to investigate structures with dimensions close to the system’s diffraction limit. The measured values agree with the known sample information and provide a clear three-dimensional visualization of the sample.

## 3. Results

The LIPSS sample was investigated with our synthetic optical holographic microscope, showing the parallel periodic surface structures of the sample. A continuous-line scan along the X-axis while the controller’s Analog-Digital-Converter (ADC) recorded the photodetector (PD) signal with a frequency of f=8 kHz captured ∼3000 points per line over a distance of 60 μm at an average velocity of $v=160\frac{\mu m}{s}$ in constant sampling mode. After each line scan in the X-direction and movement to the next initial X- and Y-positions, the reference mirror PZM was translated one step further. The distance between Y-positions was 30 nm. Including the return paths to the initial X-position, movement to the next Y-line, and data gathering time, a scan of a 60 μm·60 μm area with the chosen parameters took about ∼1 h when the stages were set to the smallest step size for maximum accuracy. This scanning time could be reduced by increasing the step sizes, but the limits for resolution and sampling losses should be respected. In order to avoid scanning regions with strong acceleration and deceleration of the sample stage, only a centered region of interest of 30 μm·30 μm was used for further processing. For creating an equidistant grid of pixels, the collected data points were numerically gridded and interpolated to a matrix of pixels with a pixel size of 15 nm·15 nm as a resulting image. Compared to the optical resolution of the system, the scanning procedure was performed with much smaller steps, causing oversampling and thus preventing sampling losses.

**Figure 7:**
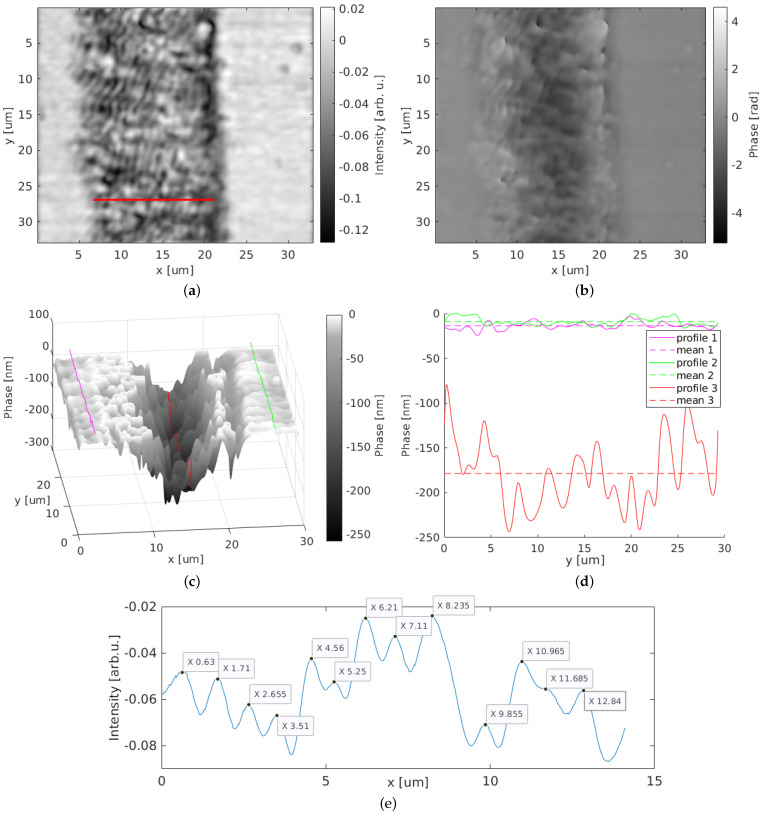
LIPSS sample, here with parallel periodic structures, imaged with the synthetic optical holographic microscope and reconstructed: (**a**) amplitude image (red line is used as example to investigate the periodicity of the structures); (**b**) phase image; (**c**) three-dimensional surface plot; (**d**) line profiles from (**c**); (**e**) line profile of red line in (**a**).

The scanned holographic image was numerically reconstructed using the aforementioned algorithms, resulting in amplitude and phase images, as shown in Figure 7a,b. The amplitude image shows a detailed overview of the periodic structures on the surface, vertically patterned along a line on the polycrystalline diamond surface and surrounded by the unprocessed flat environmental surface. The additional phase image shows the sample with a different emphasis on the LIPSS patterns, since the phase image is only sensitive to refractive index and height variations and not to the back-reflection intensities such as the amplitude.

By taking the red horizontal line at position y=27 μm of the amplitude image (Figure 7a), the line profile was extracted and is shown in Figure 7e. The peaks of the laser-induced periodic surface structures visible in this line profile show an average period length of 1.01 μm with a standard deviation of 0.24 μm. This value is 182 nm above the average period length measured with AFM and thus within the standard deviation. Further variations may be introduced in the sample during fabrication of the LIPSSs. For the comparison of AFM and holographic images, due to problems in accurate determination of the AFM imaging area, we could not ensure that exactly the same sample position was monitored. Thus, we attribute the difference in the average periodicity values to the different sample region and structure orientations.

Figure 7c shows the LIPSS sample in a three-dimensional topographic surface image derived from the reconstructed phase acquired with the confocal holographic laser-scanning setup, as well as the three extracted line profiles; one is from the structure valley (red) and two are from the surrounding sample surface (green and purple). The dashed lines indicate the corresponding mean values.

## 4. Discussion and Conclusions

The measurements of the test target demonstrates that our synthetic optical holographic microscope was capable of imaging structures down to the confocally enhanced diffraction limit of the system with high lateral and axial accuracy. Its phase imaging capabilities were tested and shown for structures of a 120 nm height. Generally, phase structures in the range of the wavelength (half the wavelength, considering the reflection geometry) can be investigated due to the 2π phase ambiguity. However, measurements above this range are possible if needed and can be enabled by additionally applying a phase-unwrapping algorithm during reconstruction. All together, the acquisition of amplitude and phase images, as well as three-dimensional topographic surface plots, is presented. The limits of the system occur when imaging sharp edges over small lateral dimensions due to the limited modulation transfer function. Nevertheless, the system was able to distinguish 600 nm structures, which are slightly below its theoretical diffraction limit of 611 nm. The diffraction-limited resolution of the system could be further improved using a shorter wavelength in the blue or even UV wavelength range in order to investigate smaller structures, if needed.

The investigation of the presented LIPSS sample, performed with the SOH microscope, shows the system’s capability to three-dimensionally image laser-induced periodic surface structures. The lateral dimensions of both amplitude and phase agree well with the expectations from sample fabrication as well as with the comparison measurements. The heights measured with the holographic setup vary in respect to the heights measured with the AFM, although they were both in the same order of magnitude. Generally, holographic setups such as our SOH are sensitive to both height variations and changes in the complex refractive index in the studied materials. On the one hand, this can make the data interpretation more complex, but on the other hand, it can provide additional information about the changes in the chemical composition of the sample due to e.g., laser-induced chemical reactions or changes in the oxygen concentration. However, in our case, the difference between the holographically measured phase and the structure height measured with the AFM is most probably due to the different sample region with different LIPSS orientation caused by the limited availability of sample access and comparison AFM data. Furthermore, it is expected that the sample itself shows height variations, introduced during the fabrication process. The resolution of the system might also be a limiting factor for the quantitative height measurement, since the investigated LIPSS patterns with sizes in the range 830–1010 nm are close to the resolution of the imaging system with a resolution limit of 611 nm. Although the system is proven to be capable of imaging the given structures with a high lateral resolution as well as accurate phase sensitivity, the height detection of structures close to the resolution limit is also limited by the modulation depth. This boundary of the modulation transfer function could restrain the investigable heights over small lateral dimensions. However, this explains the height differences in the periodic patterns, but not in the mean depth of the overall structure valley.

In addressing this issue, another phenomenon might play a significant role in variation in height. The processing of polycrystalline diamond substrates using laser pulses may create graphitic layers on the surface of the sample in the order of hundreds of nanometers, as has been reported in [39], as well as in the amount of ∼165 nm in [40]. It is not yet investigated how such additional surface layers could influence the holographic and AFM measurements. However, combining SOH with spectrally dependent measurements could be a promising tool for future studies to obtain additional information about such surface layers and their chemical compositions. Furthermore, the investigation of LIPSSs underneath the surface [41] using SOH bears great potential for future applications, since holographic techniques are potentially capable of performing subsurface imaging, as has been shown for buried micro-circuits [28].

Though the investigation of these issues that are outside the scope of this paper should be further analyzed, it can be emphasized that quantitative lateral measurement, as well as surface topography measurement, can be performed well with the synthetic optical holographic microscope. The reconstructed amplitude and phase images can be used to three-dimensionally image samples and derive the shape and periodicity of LIPSSs.

In summary, it is here shown that three-dimensional investigation of laser-induced periodic surface structures on a polycrystalline diamond substrate can be performed with a synthetic optical holographic microscope with a diffraction-limited lateral resolution of ∼600 nm. The measurement was performed without any preparation, processing of, or mechanical contact with the sample at all, and was entirely non-destructive and kept the sample fully intact. The reconstruction of the recorded hologram provided amplitude and phase images as well as a three-dimensional image of the sample by only measuring one single layer. The resulting images showed high lateral accuracy and a surface topography, which can be used to identify the dimensions, shape, and periodicity of LIPSSs.

## Data Availability

The data presented in this study are available from the corresponding author on reasonable request.

## References

[1] Emmony D.C., Howson R.P., Willis L.J. (1973). Laser mirror damage in Germanium at 10.6 μm. Appl. Phys. Lett..

[2] Isenor N.R. (1977). CO_2_ laser-produced ripple patterns on Ni_*x*_P_1-*x*_ surfaces. Appl. Phys. Lett..

[3] Siegrist M., Kaech G., Kneubühl F. (1973). Formation of a periodic wave structure on the dry surface of a solid by TEA-CO2-laser pulses. Appl. Phys..

[4] Birnbaum M. (1965). Semiconductor Surface Damage Produced by Ruby Lasers. J. Appl. Phys..

[5] Costache F., Kouteva-Arguirova S., Reif J. (2004). Sub–damage–threshold femtosecond laser ablation from crystalline Si: Surface nanostructures and phase transformation. Appl. Phys. A.

[6] Costache F., Henyk M., Reif J. (2002). Modification of dielectric surfaces with ultra-short laser pulses. Appl. Surf. Sci..

[7] Dusser B., Sagan Z., Soder H., Faure N., Colombier J.P., Jourlin M., Audouard E. (2010). Controlled nanostructrures formation by ultra fast laser pulses for color marking. Opt. Express.

[8] Liu H., Lin W., Hong M. (2019). Surface coloring by laser irradiation of solid substrates. APL Photonics.

[9] Dostovalov A., Bronnikov K., Korolkov V., Babin S., Mitsai E., Mironenko A., Tutov M., Zhang D., Sugioka K., Maksimovic J. (2020). Hierarchical anti-reflective laser-induced periodic surface structures (LIPSS) on amorphous Si films for sensing applications. Nanoscale.

[10] Bialuschewski D., Hoppius J.S., Frohnhoven R., Deo M., Gönüllü Y., Fischer T., Gurevich E.L., Mathur S. (2018). Laser-Textured Metal Substrates as Photoanodes for Enhanced PEC Water Splitting Reactions. Adv. Eng. Mater..

[11] Qian Y., Huang H., Wang C., Yu P., Xu J., Zhang Z. (2021). Formation of leaf-shaped microstructure on Zr-based metallic glass via nanosecond pulsed laser irradiation. J. Manuf. Process..

[12] Wang C., Huang H., Qian Y., Zhang Z., Huang W., Yan J. (2022). Nitrogen assisted formation of large-area ripples on Ti6Al4V surface by nanosecond pulse laser irradiation. Precis. Eng..

[13] Bonse J., Kirner S.V., Höhm S., Epperlein N., Spaltmann D., Rosenfeld A., Krüger J. (2017). Applications of laser-induced periodic surface structures (LIPSS). Laser-Based Micro-and Nanoprocessing XI.

[14] Florian C., Kirner S.V., Krüger J., Bonse J. (2020). Surface functionalization by laser-induced periodic surface structures. J. Laser Appl..

[15] Hamdorf A., Olson M., Lin C.H., Jiang L., Zhou J., Xiao H., Tsai H.L. (2011). Femtosecond and nanosecond laser fabricated substrate for surface-enhanced Raman scattering. Opt. Lett..

[16] Borodaenko Y., Syubaev S., Gurbatov S., Zhizhchenko A., Porfirev A., Khonina S., Mitsai E., Gerasimenko A.V., Shevlyagin A., Modin E. (2021). Deep Subwavelength Laser-Induced Periodic Surface Structures on Silicon as a Novel Multifunctional Biosensing Platform. ACS Appl. Mater. Interfaces.

[17] Zhou W., Apkarian R., Wang Z.L., Joy D. (2006). Fundamentals of scanning electron microscopy (SEM). Scanning Microscopy for Nanotechnology.

[18] Phillips H.M., Callahan D.L., Sauerbrey R., Szabo G., Bor Z. (1992). Direct laser ablation of sub-100 nm line structures into polyimide. Appl. Phys. A.

[19] Kirner S.V., Wirth T., Sturm H., Krüger J., Bonse J. (2017). Nanometer-resolved chemical analyses of femtosecond laser-induced periodic surface structures on titanium. J. Appl. Phys..

[20] Cangueiro L.T., Cavaleiro A.J., Morgiel J., Vilar R. (2016). Mechanisms of the formation of low spatial frequency LIPSS on Ni/Ti reactive multilayers. J. Phys. D Appl. Phys..

[21] Zhang F., Nie Z., Huang H., Ma L., Tang H., Hao M., Qiu J. (2019). Self-assembled three-dimensional periodic micro-nano structures in bulk quartz crystal induced by femtosecond laser pulses. Opt. Express.

[22] Neale A.R., Jin Y., Ouyang J., Hughes S., Hesp D., Dhanak V., Dearden G., Edwardson S., Hardwick L.J. (2014). Electrochemical performance of laser micro-structured nickel oxyhydroxide cathodes. J. Power Sources.

[23] Mustafa H., Mezera M., Matthews D., Römer G. (2019). Effect of surface roughness on the ultrashort pulsed laser ablation fluence threshold of zinc and steel. Appl. Surf. Sci..

[24] Schnell M., Carney P., Hillenbrand R. (2014). Synthetic optical holography for rapid nanoimaging. Nat. Commun..

[25] Schnell M., Perez-Roldan M., Carney P., Hillenbrand R. (2014). Quantitative confocal phase imaging by synthetic optical holography. Opt. Express.

[26] Schnell M., Buercklin S., Sarriugarte P., Perez-Roldan M., Hillenbrand R., Carney P. (2016). Synthetic holographic phase imaging in confocal microscopy and applications. Frontiers in Optics.

[27] Canales-Benavides A., Zhuo Y., Amitrano A.M., Kim M., Hernandez-Aranda R.I., Carney P.S., Schnell M. (2019). Accessible quantitative phase imaging in confocal microscopy with sinusoidal-phase synthetic optical holography. Appl. Opt..

[28] Schnitzler L., Neutsch K., Schellenberg F., Hofmann M.R., Gerhardt N.C. (2021). Confocal laser scanning holographic microscopy of buried structures. Appl. Opt..

[29] Zhang B., Liu S., Wu X., Yi T., Fang Y., Zhang J., Zhong Q., Peng X., Liu X., Song Y. (2017). Ultrafast dynamics of carriers and nonlinear refractive index in bulk polycrystalline diamond. Optik.

[30] Kiel F., Bulgakova N.M., Ostendorf A., Gurevich E.L. (2019). Selective Delamination upon Femtosecond Laser Ablation of Ceramic Surfaces. Phys. Rev. Appl..

[31] Anzolin G., Gardelein A., Jofre M., Molina-Terriza G., Mitchell M.W. (2010). Polarization change induced by a galvanometric optical scanner. JOSA A.

[32] Udupa G., Singaperumal M., Sirohi R., Kothiyal M. (2000). Characterization of surface topography by confocal microscopy: I. Principles and the measurement system. Meas. Sci. Technol..

[33] Pawley J. (2006). Handbook of Biological Confocal Microscopy.

[34] Kim M.K. (2011). Digital Holographic Microscopy.

[35] Barer R. (1952). Interference microscopy and mass determination. Nature.

[36] Bhaduri B., Edwards C., Pham H., Zhou R., Nguyen T.H., Goddard L.L., Popescu G. (2014). Diffraction phase microscopy: Principles and applications in materials and life sciences. Adv. Opt. Photonics.

[37] Hecht E. (2018). Optik.

[38] Cox G., Sheppard C.J. (2004). Practical limits of resolution in confocal and non-linear microscopy. Microsc. Res. Tech..

[39] Okuchi T., Ohfuji H., Odake S., Kagi H., Nagatomo S., Sugata M., Sumiya H. (2009). Micromachining and surface processing of the super-hard nano-polycrystalline diamond by three types of pulsed lasers. Appl. Phys. A.

[40] Su S., Li J., Lee G.C., Sugden K., Webb D., Ye H. (2013). Femtosecond laser-induced microstructures on diamond for microfluidic sensing device applications. Appl. Phys. Lett..

[41] Rudenko A., Colombier J.P., Höhm S., Rosenfeld A., Krüger J., Bonse J., Itina T.E. (2017). Spontaneous periodic ordering on the surface and in the bulk of dielectrics irradiated by ultrafast laser: A shared electromagnetic origin. Sci. Rep..

